# A Simple Modification of the Conventional Figure-of-Eight Sternal Closure Technique

**DOI:** 10.21470/1678-9741-2018-0244

**Published:** 2019

**Authors:** Carlos Junior Toshiyuki Karigyo, Aldo Pesarini

**Affiliations:** 1 Hospital Norte Paranaense (HONPAR), Arapongas, PR, Brazil.; 2 Hospital do Câncer de Cascavel (UOPECCAN), Filial Umuarama, PR, Brazil.; 3 Instituto Nossa Senhora Aparecida (INSA), Umuarama, PR, Brazil.; 4 Universidade Paranaense (UNIPAR), Umuarama, PR, Brazil.

**Keywords:** Sternum - Surgery, Mediastinitis, Steel, Bone Wires, Hospital Mortality, Wound Closure Techniques

## Abstract

**Objective:**

To describe a new technique of sternal closure, modified from the conventional figure-of-eight approach, which can provide a secure closure and prevent sternal complications.

**Methods:**

The modified technique is based on the intercalation of the caudal portion of each steel wire passed along the sternum. This is a retrospective analysis of patients operated with this modified technique at our institution between January 2014 and December 2016.

**Results:**

One hundred and forty-three patients underwent sternal closure with the modified technique. In-hospital mortality rate was 1.4% (n=2). No sternal instability was observed at 30 days postoperatively. Two patients developed mediastinitis that required extraction of the wires.

**Conclusion:**

Short-term results have shown that the modified sternal closure technique can be used safely and effectively, with complications rates being consistent with worldwide experience.

**Table:** 

Abbreviations, acronyms & symbols	
ASD	= Atrial septal defect
CABG	= Coronary artery bypass grafting
EuroSCORE	= European System for Cardiac Operative Risk Evaluation
NYHA	= New York Heart Association
SD	= Standard deviation
VSD	= Ventricular septal defect

## INTRODUCTION

Sternal closure after median sternotomy can be performed by a wide range of techniques, but there is still a lack of high-quality evidence supporting an optimal procedure^[1]^. During decades, cardiothoracic surgeons have employed numerous approaches and their respective modifications to prevent sternal complications, applying multiple wiring techniques such as implanting different materials and devices^[2]^. Even with some evidence suggesting that implantable device systems may be advantageous compared to the traditional closure with wires alone^[3,4]^, their application fails because of a mismatch in practicability and financial support^[5,6]^. Alternatively, many wiring techniques were conceived and evaluated, including transsternal and parasternal approaches, and a more stable closure has been reported when oblique tension on the sutures and some form of lateral support were applied^[7,8]^. However, no similar technique to that used in our unit seems to combine all together the previously mentioned tension and supporting characteristics within a feasible manner.

We present in this article our clinical experience with this technical modification, conceived originally from the conventional figure-of-eight approach, which does not require additional material, provides secure and effective closure by preventing sternal displacement, and allows easy reentry if necessary. We hypothesized that the obliquely distributed tension along the sternal table confers a more stable fixation, preventing sternal movement and separation, reducing the risks for dehiscence.

## METHODS

This is a retrospective study and the protocol was approved by the Research Ethics Committee from the Universidade Paranaense - UNIPAR, Umuarama, Paraná, Brazil (under the number 2.679.020), and registered at the Plataforma Brasil (CAAE 89661718.0.0000.0109). Due to the retrospective nature of the study, the need for individual patient consent was waived.

### Patients

Data were collected from medical records of all cardiac surgical patients who underwent the modified sternal closure technique by a single senior surgeon (AP), from January 2014 to December 2016, at a single institution (Instituto Nossa Senhora Aparecida - INSA, Umuarama, Paraná, Brazil).

Sternal stability was assessed from postoperative physical examination records reporting the absence of sternal movement (undetectable displacement by palpation during coughing or with Valsalva maneuver), while instability was considered in the presence of an apparent crepitation or slight displacement until a complete sternal separation.

### Statistical Analysis

Collected data were analyzed by descriptive statistics, with continuous variables being expressed as mean ± standard deviation (SD) and categorical variables expressed as absolute numbers and percentages (%). The storage and analysis of the data were done in the Microsoft Excel^®^ 2013 software.

### The Modified Sternal Closure Technique

The modified technique was originally conceived in 2005. Since then, it has been routinely applied in our unit as a result of a perceived very-low complication rate and a robust reduction in the need for opioid analgesia (Pesarini A, personal communication).

### Description

**Fig. 1:**
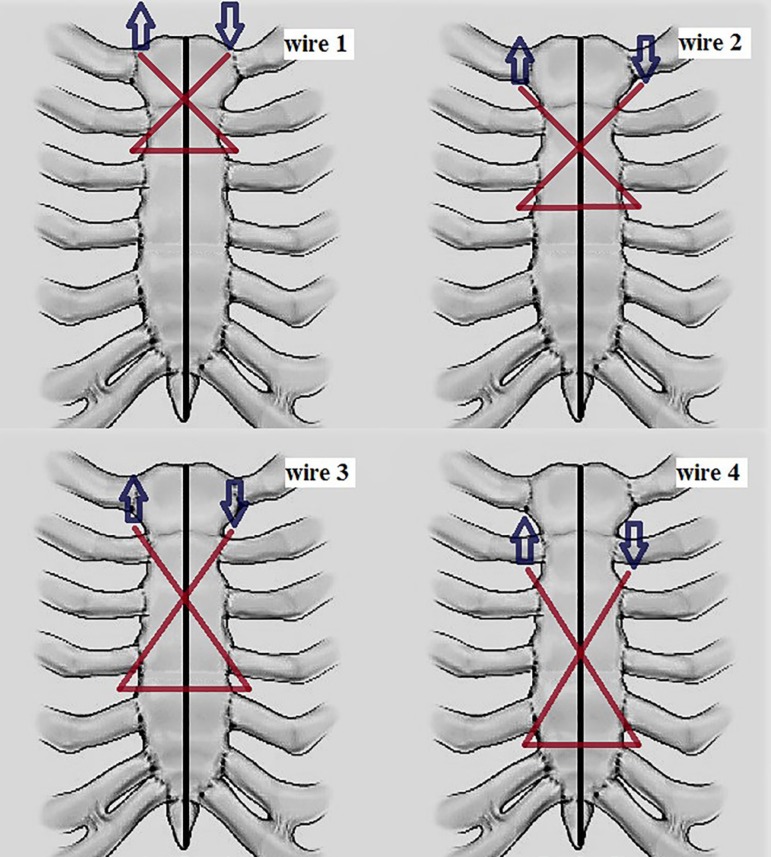
Schematic representation of the modified technique. Arrows indicate the direction of each wire (1-4), as previously described.

**Fig. 2:**
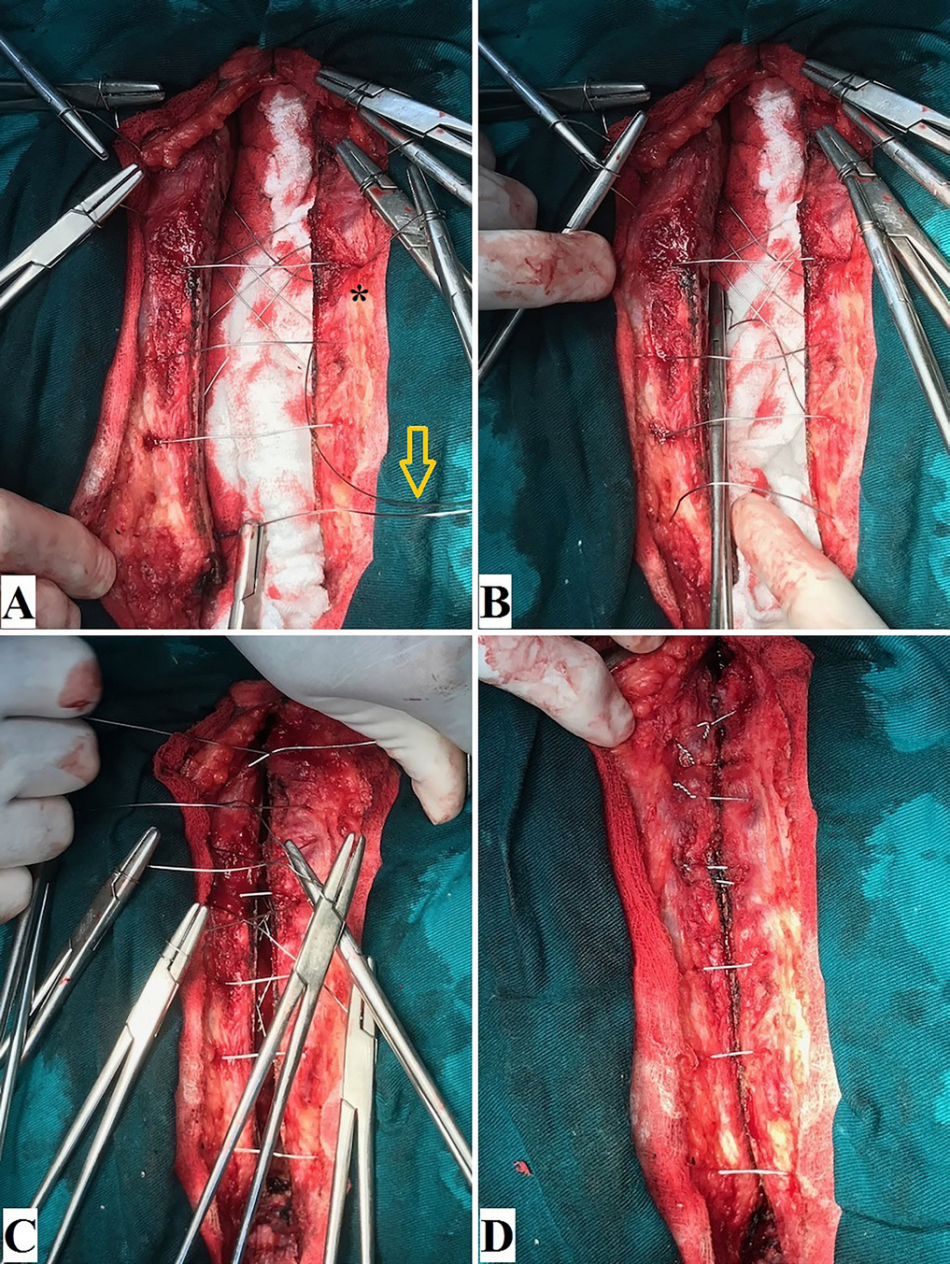
Modified sternal closure technique: (A-B) fourth wire (arrow) being placed (asterisk indicates the fourth wire’s entry); (C) reapproximating the sternal edges; (D) final aspect, with the knots placed on the top of the sternal table.

We use four stainless steel wires for sternal closure. Wire 1 is applied transternally through the manubrium, near the 1^st^ rib attachment at left, crossing the 2^nd^ intercostal space on both sides, then returning to the right edge of the manubrium. Wires 2 and 3 are applied through the 1^st^ intercostal space and the sutures are completed crossing the wires through the 3^rd^ and the 4^th^ intercostal spaces, respectively. Wire 4 is applied through the 2^nd^ intercostal space and crosses caudally the 5^th^ intercostal space (Figures 1 and 2).

## RESULTS

A total of 143 cardiac operations performed with the modified sternal closure technique were conducted on the period of the study. Closure was performed by a single senior surgeon (AP). The demographic and clinical characteristics of the patients are summarized in Table 1.

**Table 1:** Patients' clinical profile and demographics.

Demographics and types of surgeries	Range	*n* (%)
Sex	Female (56)	Total = 143
	Male (87)	
Age	[1-79] years	52.1 ± 20.3 years
Hypertension		78 (54.5%)
Diabetes	Non-insulin-dependent (19)	29 (20.3%)
	Insulin-dependent (10)	
Chronic renal disease	Non-dialytic (5)	8 (5.6%)
	Dialytic (3)	
Heart failure	NYHA class III-IV (10)	11 (7.7%)
	Cardiogenic shock (1)	
Smoking		14 (9.8%)
Severe pulmonary disease		5 (3.5%)
Morbid obesity		5 (3.5%)
Peripheral arterial occlusive disease		2 (1.4%)
Pulmonary hypertension	Congenital (21)	47 (32.8%)
	Adult (26)	
CABG	Elective (71)*	72 (50.3%)
	Emergency (1)	
Valvular	Mitral (16)	39 (27.3%)
	Aortic (11)	
	2 or + valves (12)	
Congenital	ASD (16)	21 (14.7%)
	ASD + VSD (3)	
	Subaortic membrane (2)	
Combined	Valvular + CABG (3)	10 (7%)
	Valvular + ascending aorta (7)	
Pericardiectomy (tamponade)		1 (0.7%)

ASD=atrial septal defect; CABG=coronary artery bypass grafting; NYHA=New York Heart Association; VSD=ventricular septal defect

* Including 1 reoperation

There were no bleeding complications related to sternal wiring. Reoperation for hemorrhage occurred in one patient due to a stitch rupture in the ascending aorta, with a good postoperative outcome. In-hospital mortality rate was 1.4% (two patients), one patient died from cardiogenic shock and the other from sepsis of pulmonary origin. Clinical examination confirmed sternal stability in all patients at 30 days postoperatively. Two patients (*n*=2 or 1.4%) developed mediastinitis along hospitalization and required the removal of all wires in association with antibiotics administration. After resolution of the infection, one patient required surgical reconstruction using Robicsek’s technique and the other using pectoral muscle advancement flap for reclosure. Indeed, both patients were insulin-dependent diabetics and one of them was morbidly obese. None of them died.

## DISCUSSION

Median sternotomy was introduced in the 1950s. It is currently the preferred choice for sternal incision in cardiac surgery and remains the most common osteotomy, being performed in more than 500.000 patients/year in the United States alone^[2,3]^. Despite another established approaches usually performed in thoracic surgical procedures such as lateral thoracotomy, median sternotomy still remains the favorite procedure because it is easy to execute and provides excellent exposure, being also associated with less respiratory complications and pain^[2,5]^.

Closure of median sternotomy with wires has been the gold-standard technique for more than 50 years for being considered easily and quickly achievable, reproducible with a perceived low complication rate, and cheap because of the low-cost of steel wires^[3,6]^. Nevertheless, mediastinitis is a rare complication but can be devastating and may represent clinical and economical challenges, with incidence rates varying from 0.5% to 5% and mortality rates ranging between 14% and 47%^[2,9-11]^. Advanced age, diabetes, obesity, renal failure, chronic pulmonary disease, osteoporosis, and poor nutritional status are among the predisposing factors, and many alternatives have emerged in order to avoid sternal wound complications^[2-6,9-11]^. Although an increasing number of devices, such as rigid plates, cables, and titanium hooks, seemed to become superior to the traditional method with a limited amount of evidence^[3-6,12]^, sternal complications still occurred with all of these alternatives^[4,13]^.

There is a wide range of sternal closure techniques applying only steel wires justified by the still unconvincing evidence for device incorporation. In addition to the anchoring-based Robicsek’s technique and its derived modifications, usually applied to complicated sternal closures^[14-16]^, some authors proposed multiple types of approaches based on diverse wiring methodology. Casha et al.^[17]^ published an alternative method with interlocking multitwisted wires to enhance sternal stabilization and minimize bleeding from sternal fractures. They routinely applied eight wires along the suture and used this technique in more than 2000 patients over a 10-year period with a dehiscence rate of 0.5%. Saxena et al.^[18]^ proposed the use of double wires for sternal closure in obese patients, with a reduction in sternal dehiscence rate to approximately 1%. The authors applied seven stainless steel wires to perform the fixation of the sternum, reporting occasional bleeding from the chest wall consequent to the double penetration in the intercostal spaces. Konstantinov et al.^[19]^ described an interlocking wiring technique introduced in 25 consecutive high-risk patients, with no sternal dehiscence. It should be clear, indeed, that sternal dehiscence is intrinsically related to mediastinitis.

According to Kamiya et al.^[20]^, the number of wires applied has also a significant influence on preventing sternal complications, which occurred in 2.4% of all patients from their study. In addition, the use of less than eight wires was significantly associated with postoperative sternal complications in the high-risk group. Jolly et al.^[21]^ described what they called “the butterfly technique”, which required twelve steel wires to perform sternal closure. The technique was based on twisting two conjoined wires on the same side, forming a cable, and then twisting these cables with the opposite ones to form a final cabled knot. They routinely applied such technique in 291 patients with no sternal dehiscence or wound healing problems.

Although these approaches may prevent sternal complications with excellent outcomes, they demand an addition on the wires counting and may be more labor-intensive and increase the surgical time than the standard method. We agree that the key factor in preventing sternal dehiscence lies in a stable sternal approximation. But the incorporation of more content - in wires or in any type of devices - would not be mandatory to guarantee the stability of a sternal fixation^[22,23]^. In the contrary, an additional material in the sternum could turn an effortless emergent reentry into a tumultuous surgical procedure.

Khasati et al.^[24]^ demonstrated that the conventional figure-of-eight had no significant advantage over the simple wiring technique. Pai et al.^[25]^ compared the stability provided by sternal plates with standard wires using an in vitro model, concluding that rigid plates increased stability in the midline compared with wires. Considering these facts, our modified technique approximates the two sternal halves by tightening them both longitudinally than transversally, dissipating intrathoracic pressure across a broadened and firmly closed area, very differently from the conventional figure-of-eight and the simple wiring techniques. We think that this feature may confer a similar stability provided by rigid plating, bringing up a new concept of a broad-based sternal closure using only stainless steel wires.

### The Modified Technique

**Fig. 3:**
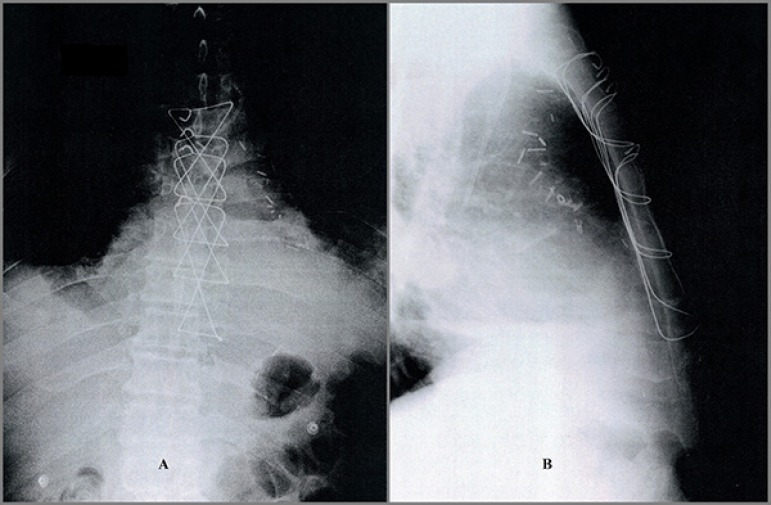
(A) Frontal and (B) lateral views on chest X-ray.

**Fig. 4:**
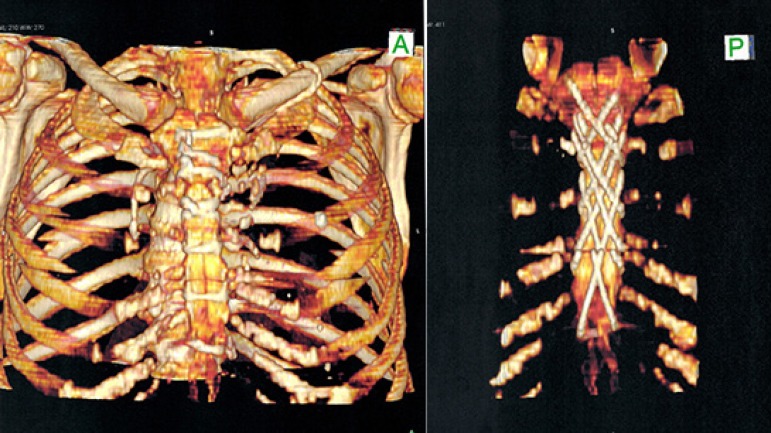
Chest computed tomography showing (A) anterior and (P) posterior aspects of the sternum closed by the technique.

The modified technique we present in this paper comprises a simple changing from the figure-of-eight wiring configuration in order to distribute oblique tension and minimize transversal bone cutting compared to the traditional way. The distributed form of the wires through the sternum also confers a more rigid and stable fixation even with the employment of no more than four steel wires, an advantage of this modification over the abovementioned techniques. Imaging studies show how the steel wires final mesh appear after sternal closure (Figures 3 and 4). Although posterior view may give us the false impression of crossed wires, they are placed parallel and consecutively one over the other. This feature enables an easy and rapid extraction in an event of emergent reexploration, as occurred with one of our patients.

The ideal method for sternal fixation should avoid complications by preventing sternal movement and separation, offer easy feasibility and reproducibility by optimizing operative time, allow rapid reentry in the event of emergent reexploration, and should be cost-effective. All of these requirements are summarized in Table 2, with comments on the modified technique presented in this paper.

**Table 2:** Characteristics of the modified technique according to established criteria and purpose. Adapted from Alhalawani et al.^[2]^.

Criteria	Purpose	Comments on the modified technique
Mechanical properties	Resistance during coughing	√ Tension is distributed
Radiopacity	Diagnosing displacement	√ Radiopaque (steel wire)
Biocompatibility	Avoiding infection, rejection, or inflammation	√ No additional material
Handling properties	Optimizing operative time	√ Quickly achievable
Removable when necessary	Facilitating extraction	√ Quick and simple maneuver (cut and pull)
		√ No device to complicate extraction
Cost-effective	To avoid limitations on use and supply	√ Same material
		√ No additional cost

### Study Limitations

Our study has some limitations that should be highlighted. This is a single-center retrospective study with a relatively small sample size and without a control group for a comparative analysis, because the modified technique is routinely employed at our unit since 2005. As another study limitations, logistic European System for Cardiac Operative Risk Evaluation (EuroSCORE), cardiopulmonary bypass time, operative time, and body mass index were not analyzed due to the lack of information in our database.

## CONCLUSION

A vast number of technical innovations have improved sternal closure outcomes, but until now there is no ideal procedure for an effective and robust sternal fixation. The appropriate technique should harmoniously contemplate several requirements, ranging from mechanical properties, operability, reproducibility, and even economical aspects. We believe that our alternative procedure fulfills most of these requirements. The modified technique is a safe, effective, and easily reproducible method for preventing sternal dehiscence.

**Table:** 

Authors' roles & responsibilities	
CJTK	Substantial contributions to the conception or design of the work; or the acquisition, analysis, or interpretation of data for the work; agreement to be accountable for all aspects of the work in ensuring that questions related to the accuracy or integrity of any part of the work are appropriately investigated and resolved; final approval of the version to be published
AP	Substantial contributions to the conception or design of the work; or the acquisition, analysis, or interpretation of data for the work; agreement to be accountable for all aspects of the work in ensuring that questions related to the accuracy or integrity of any part of the work are appropriately investigated and resolved; final approval of the version to be published
